# A Clean, Well-Lighted Place^1^

**DOI:** 10.3201/eid1604.AC1604

**Published:** 2010-04

**Authors:** Polyxeni Potter

**Affiliations:** Centers for Disease Control and Prevention, Atlanta, Georgia, USA

**Keywords:** Art science connection, emerging infectious diseases, art and medicine, Edward Hopper, Drug Store, drugs, architecture, Clostridium difficile, about the cover

**Figure:**
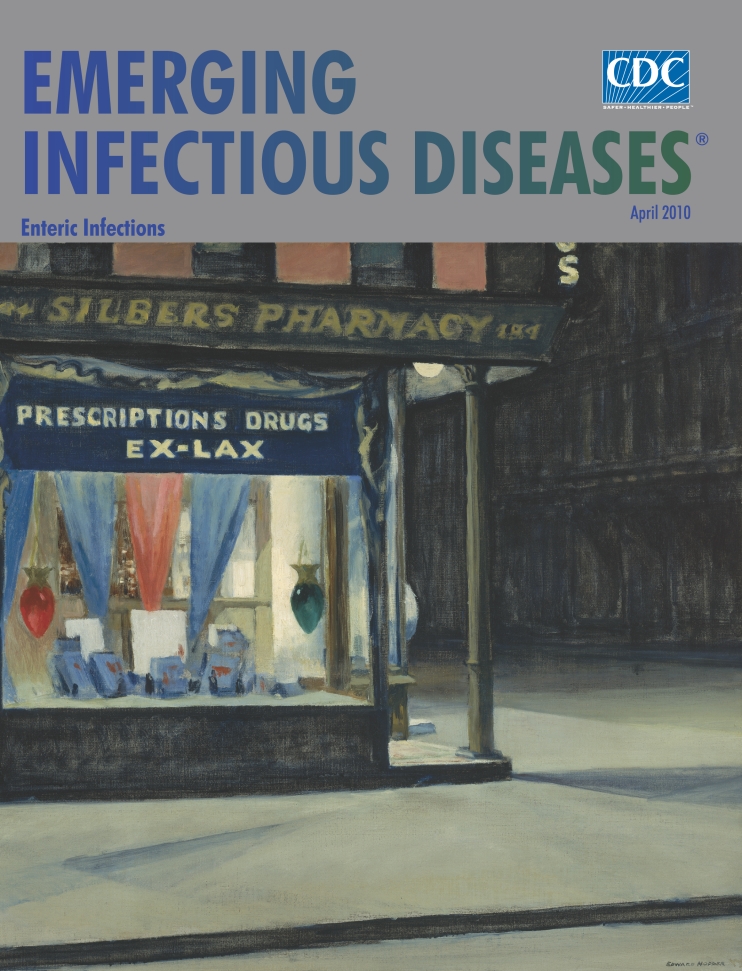
**Edward Hopper (1882–1967) Drug Store (1927) Oil on canvas** (73.6 cm × 101.9 cm) The Museum of Fine Arts, Boston, Massachusetts, USA Bequest of John T. Spaulding, 48.564

“The man’s the work. Something doesn’t come out of nothing,” Edward Hopper once said. This private and introspective man, known for his dry wit and “monumental silences” and for expressing himself “tersely but with weighted exactness in a slow reluctant monotone,” was offering a glimpse into the creative process as it applied to him. Much was made of the sense of isolation and despair in his work and their connection with modern life. But “The loneliness thing is overdone,” he noted. “My aim in painting is always, using nature as the medium, to try to project upon canvas my most intimate reaction to the subject as it appears when I like it most; when the facts are given unity by my interest and prejudices.”

“Hudson River Dutch” is how Hopper described his ancestry in Nyack, New York. The son of a dry goods merchant, he was not discouraged in his artistic ambitions, though his family did steer him toward commercial illustration for its earning potential. The skill stood him in good stead during the lean years. He attended the New York School of Art and studied under Robert Henri, one of the fathers of American Realism, “the most influential teacher I had.” He visited Europe several times. “Paris had no great or immediate impact on me.” But when he returned to the United States, his work reflected what he had seen abroad. “It took me ten years to get over Europe.”

Hopper settled in New York, where he would make his primary residence. He moved into a 74-step, cold-water walk-up with a sky-lit studio in Greenwich Village. He had to haul coal for the furnace up four flights of stairs, but the space suited his self-sufficient and frugal nature. He painted many major works there and, despite a reasonable measure of success during his lifetime, never pursued more plush quarters.

During a career that spanned 60 years and saw the heyday of abstract expressionism, as well as a resurgence of realism in the United States, he made a unique contribution. In a 1932 exhibition at the Museum of Modern Art, his work was described as “part of a new international progressive trend emerging within modernism, represented by a balance between ‘form’ and ‘content’ in its work.” Hopper’s approach, which appealed to his colleagues from all factions, explored natural and artificial light on surfaces, particularly on the vernacular architecture of American cities: motels, gas stations, storefronts, diners, apartments. “What I wanted to do was to paint sunlight on the side of a house.” His work employed classical elements and formal discipline in the dispassionate presentation of everyday scenes.

Hopper captured what he called “our native architecture with its hideous beauty, its fantastic rooms, pseudo-gothic, French Mansard, Colonial, mongrel or what not, with eye searing color or delicate harmonies of faded paint, shouldering one another along interminable streets that taper off into swamps or dump heaps,” and by capturing it, he defined it. His images, often described as theatrical or cinematic, went on to inspire the cinema and its greats, among them John Huston, Elia Kazan, John Cassavetes. Alfred Hitchcock credited Hopper for influencing his films Rear Window and Psycho*.*

“The whole answer is there on the canvas,” Hopper said in lieu of explaining his paintings. “I hope it will not tell any obvious anecdotes since none are intended.” Just like the content of his paintings, the form was stripped of extraneous details. He sought perfection in perspective, geometric structures, and two-dimensional space, as well as in the use of color and light. “As a child I felt that the light on the upper part of a home was different from that on the lower part. There is a sort of elation about sunlight on the upper part of a house.” He described his style as “an amalgam of many races” and refused to belong to any school.

“Hopper is always on the verge of telling a story,” observed novelist and art critic John Updike, referring to scenes whose very stillness suggests that something is about to happen. A room seems either recently vacated or soon to be occupied. If inhabited, it is always sparsely, and that also goes for public places―theaters, restaurants, offices. Any occupant has either just arrived or is ready to leave, psychologically absent, or lost in thought. Human presence is not required, although the viewer is always allowed in, either through an open window or some other vista. And the night is just as paintable as the day, or even more so because of its mysterious, even ominous, contrasts and shadows.

Drug Store, on this month’s cover, is one of Hopper’s nocturnal works. During one of his visits to Europe, he saw Rembrandt’s Night Watch, “The most wonderful thing…. It almost amounts to deception.” In his own work, he came to view the night as an opportunity to scrub a scene from the hustle and bustle. In the darkness, it seems, he could focus on the unexceptional and familiar elements of the uninhabited streets and capture the essence of places.

In Drug Store, drama resides strictly on the weight of darkness in back of the setting against the brightly lit establishment forward. The lamp above the entrance lights the window, which with the awning pulled up all the way, is unabashedly exposed. Emotion is rendered in place and time, not human terms. In painting this street icon, Hopper as always, sought the “most exact transcription possible.” But what might have been a most sterile, even disturbing, presentation is rendered here with softness and calm. The storefront glows against the surroundings, its curtains and colorful vessels inviting and homelike but for the commercial signage. The wedgelike positioning foreshadows another one of Hopper’s famous corner establishments, Nighthawks.

Silbers Pharmacy was typical of the profession in the early part of the 20th century. The move away from artisan plasters, powders, and carbonated waters saw increasingly flamboyant advertising of medications based on better understanding of disease etiology and the mechanisms of drug action.

These new drugs were a mixed bag of cure and trouble, just like today’s offerings. In this journal issue alone, countless examples underline unintended consequences in the use of otherwise effective medications, among them antimicrobial drugs. Commonly used to treat infections, these drugs also may change intestinal microflora and make patients vulnerable to other infections, as with *Clostridium difficile*. In addition to increased *C. difficile*–associated disease, severity is also increasing from a new strain. And while clinical illness used to occur almost exclusively among the elderly in healthcare facilities, it is now seen in the community, among the young, and apart from antimicrobial drug treatment. Some retail meats contain the pathogen, though its role there is unknown. Changes in disease setting alter who is at risk and what the risk factors are. The clean, well-lighted healthcare setting where antimicrobial drug use leads to pseudomembranous colitis does not describe the risk for or characteristics of community-acquired infections with *C. difficile*.

“I was never able to paint what I set out to paint,” Hopper said. During the unpredictable course of the creative process, inspiration injects itself unawares, the miscellaneous and extraneous intercede, color dictates, or thought transforms the initial intention to an unrecognizable final result. The artist’s discerning eye must complete the process by honing in on the essential. Public health scientists at work to stay ahead of pathogens also come up against unpredictability in the creative process. Much like Hopper, they must have an eye for the setting. One is not like another, and light makes all the difference. And while the whole answer may indeed be there on the canvas, for Hopper the essence was found in the light, whereas for the scientist trying to capture and define risk factors for infection, the essence may still be lurking in the shadows.
